# Supplementary material for: Glucose-containing vs. glucose-free dialysate for patients with maintenance hemodialysis: Study protocol for a multicenter randomized controlled study-GLUMO study
Source: PLoS One. 2025 Aug 11;20(8):e0330155. doi: 10.1371/journal.pone.0330155 (PMC12338788; doi:10.1371/journal.pone.0330155)
Supplement: S1 File — (DOCX) [file pone.0330155.s001.docx]

|  | Study Centers (Chinese) | Study Centers (English) |
| --- | --- | --- |
| 1 | 四川大学华西医院 | West China Hospital, Sichuan University |
| 2 | 西南医科大学附属医院 | The Affiliated Hospital of Southwest Medical University |
| 3 | 阆中市人民医院 | Langzhong People's Hospital |
| 4 | 自贡市第四人民医院 | The Fourth People's Hospital of Zigong City |
| 5 | 富顺县中医医院 | Fushun County Traditional Chinese Medicine Hospital |
| 6 | 达州市达州区人民医院 | Dazhou District People's Hospital, Dazhou City |
| 7 | 峨眉山市人民医院 | Emeishan People's Hospital |
| 8 | 广元市中医医院 | Guangyuan Traditional Chinese Medicine Hospital |
| 9 | 三六三医院 | Hospital 363 |
| 10 | 自贡市第一人民医院 | The First People's Hospital of Zigong City |
| 11 | 南部县人民医院 | Nanbu County People's Hospital |
| 12 | 金堂县第一人民医院 | Jintang County First People's Hospital |
| 13 | 成都中医药大学附属医院 | The Affiliated Hospital of Chengdu University of Traditional Chinese Medicine |
| 14 | 富顺县人民医院 | Fushun County People's Hospital |
| 15 | 崇州二医院 | Chongzhou Second Hospital |
| 16 | 隆昌市人民医院 | Longchang People's Hospital |
| 17 | 平昌县人民医院 | Pingchang County People's Hospital |
| 18 | 自贡市第三人民医院 | Zigong Third People's Hospital |
| 19 | 仁寿县人民医院 | Renshou County People's Hospital |
| 20 | 成都市第七人民医院 | Chengdu Seventh People's Hospital |
| 21 | 雅安市雨城区人民医院 | Yucheng District People's Hospital, Ya'an City |
| 22 | 九O三医院 | 903 Hospital |
| 23 | 阿坝州林业中心医院 | Aba Forestry Center Hospital |
| 24 | 资阳市中心医院 | Ziyang Central Hospital |
| 25 | 雅安市人民医院 | Ya'an People's Hospital |
| 26 | 广安市人民医院 | Guang'an People's Hospital |
| 27 | 泸州市人民医院 | Luzhou People's Hospital |
| 28 | 西南医科大学附属中医医院 | Affiliated Traditional Chinese Medicine Hospital of Southwest Medical University |
| 29 | 巴中市中心医院 | Bazhong Central Hospital |
| 30 | 攀枝花市中心医院 | Panzhihua Central Hospital |
| 31 | 攀钢集团总医院 | Pangang Group General Hospital |
