# Supplementary material for: Glucose-containing vs. glucose-free dialysate for patients with maintenance hemodialysis: Study protocol for a multicenter randomized controlled study-GLUMO study
Source: PLoS One. 2025 Aug 11;20(8):e0330155. doi: 10.1371/journal.pone.0330155 (PMC12338788; doi:10.1371/journal.pone.0330155)
Supplement: S3 File — (DOCX) [file pone.0330155.s003.docx]

**Glucose-containing vs. glucose-free dialysate for patients with maintenance hemodialysis: study protocol for a multicenter randomized controlled study-GLUMO study**

1. **Background**

With the increasing prevalence of diabetes, hypertension and obesity, an increasing number of patients would develop chronic kidney disease (CKD), with a considerable number eventually progressing to end-stage kidney disease (ESKD) and requiring renal replacement therapy [1]. Hemodialysis (HD) is the most common form of renal replacement therapy for patients with ESKD.

Currently, dialysate used in most hemodialysis facilities is glucose-free dialysate, which provides advantages such as less risk of infection and disordered blood lipid metabolism [2]. However, as type 2 diabetes (T2DM) has become a leading cause of ESKD, the risk of severe hypoglycemia (<5.55 mmol/L) during HD has become a concern. Due to issues such as impairment of renal gluconeogenesis, malnutrition, and the use of glucose-free dialysate, HD patients are more susceptible to hypoglycemia, which is associated with seizures, stroke, and higher mortality [3]. HD can independently cause hypoglycemic events in HD patients []4. As high blood flow with continuous flow of glucose-free dialysate or low glucose dialysate through dialyzer, a loss of 20-40 g of glucose would occur during each hemodialysis session, which is an important element in the occurrence of hypoglycemic events during HD [5].

Therefore, adding an appropriate amount of glucose to the dialysate can reduce the loss of glucose in the dialysate to a certain extent, make up for the lack of blood glucose in the patient during HD, and effectively reduce the incidence of hypoglycemia. The reduction in the incidence of hypoglycemia can reduce the risk of blood pressure lowering to a certain extent. Currently, dialysate containing glucose is used in most European countries, the United States, and Japan to prevent hypoglycemia [6]. Dialysate containing 5 to 10 mmol/L glucose has been shown to reduce hypoglycemia, hypotension, and heart rate variability [7,8]. The results of a prospective crossover study in China showed that the use of glucose-free dialysate and 5.55 mmol/L glucose dialysate both lowered blood glucose after dialysis, and glucose-containing dialysate could reduce the incidence of hypoglycemia and serum sodium, but had no effect on blood pressure during HD [9]. Meta-analyses have shown that 5 mmol/L or 5.5 mmol/L of glucose-containing dialysate not only increases blood glucose and prevents dialysis hypoglycemia in patients with diabetic nephropathy during HD, but also avoids hyperglycemia and parasympathetic activity, and it is recommended that medical units could be better to use glucose dialysate at concentrations close to physiological concentrations of 5 mmol/L or 5.5 mmol/L [9]. Conversely, another intervention study found that hepcidin-25 clearance was lower with 5.55 mmol/L glucose dialysate than glucose-free dialysate during a single HD episode, suggesting that hepcid-25 clearance may be affected by dialysate glucose concentration, and that glucose-containing dialysate may not be the best choice for all patients with MHD.

At present, the conclusions of the research on the glucose concentration and effect of dialysate are not uniform, and the sample size of each study is generally small, and the long-term prognostic impact on dialysis patients has not been further explored, and there is a lack of relevant high-level evidence-based evidence. This study will observe the effect of 5.5mmol/L glucose dialysate on blood glucose, blood pressure variability, hypoglycemic events, vascular access disorder events, and the incidence of major cardiovascular and cerebrovascular adverse events in ESKD patients through a multicenter, prospective, randomized controlled study, and observe the effect on 3-year mortality, aiming to evaluate the efficacy and safety of sugary dialysate, and provide new evidence for exploring the optimal dialysis treatment prescription and the establishment of guidelines for ESKD patients.

1. **Objectives**

Major objective: To assess the effects of sugar-based dialysate therapy on major adverse cardiac and cerebrovascular events (MACCE) in patients on MHD.MACCE refers to all-cause death, heart failure, recurrent myocardial infarction, cerebrovascular accident, and recurrent cardiovascular and cerebrovascular revascularization.

References: Mäkikallio T, Holm NR, Lindsay M, et al. Percutaneous coronary angioplasty versus coronary artery bypass grafting in treatment of unprotected left main stenosis (NOBLE): a prospective, randomised, open-label, non-inferiority trial. Lancet. 2016; 388(10061):2743-2752.

Secondary objective: 1) To evaluate the effect of glucose-based dialysate treatment in reducing all-cause mortality in patients with MHD;

2) To evaluate the effects of glucose-containing dialysate therapy on hypotension, hypoglycemia, vascular access dysfunction, cardiac function, fatigue in maintenance hemodialysis patients;

3) To evaluate the safety of glucose-containing dialysate therapy.

**3.** **Study design, methods, and** **procedures**

**3.1 Study design**

We are planned to adopt the method of open-label, multi-center parallel randomized controlled study, and it is planned to include more than 30 hospitals such as West China Hospital of Sichuan University and the Affiliated Hospital of Southwest Medical University. A total of 600 patients with stable ESRD who meet the inclusion criteria for dialysis age ≥ 3 months and conventional HD treatment 3 times a week will enter the study, and after signing the informed consent form, they will be stratified according to whether they have diabetic nephropathy, and the block length will be 4 or 6. Subjects were randomly assigned to glucose-containing dialysate and glucose-free dialysate in a 1:1 ratio, and relevant primary and secondary outcome data were collected, and the efficacy of glucose-containing dialysate and glucose-free dialysate in the treatment of MHD patients was compared.

Dialysate formulation: glucose-containing dialysate group (dialysate flow rate 500 ml/min, sodium 138 mmol/L, potassium 2.0 mmol/L, calcium 1.25-1.75 mmol/L, bicarbonate 33.5 mmol/L, glucose 5.5 mmol/L, temperature 35.5-37 ℃, bacterial count<100 cfu/ml, endotoxin<0.5 EU/ml). Conventional glucose-free dialysate group (dialysate flow rate 500 ml/min, sodium 138 mmol/L, potassium 2.0 mmol/L, calcium 1.25-1.75 mmol/L, bicarbonate 33.5 mmol/L, temperature 35.5-37 ℃, bacterial count<100 cfu/ml, endotoxin<0.5 EU/ml).

Dialysis prescription: maintenance HD three times per week; arteriovenous fistula (AVF), tunnel-cuffed catheter (TCC) or arteriovenous graft (AVG) as vascular access; dialysis fluid flow rate of 500ml/min; blood flow rate of 200-300ml/min; Prioritize using of high cutoff dialyzer with a membrane area of 1.4-1.8m2 and a membrane ultrafiltration coefficient over 20ml/h/mmHg; Unfractionated heparin (UFH) or low-molecular-weight heparin (LMWH) as anticoagulants.

Sample size: In this study, the sample size was calculated with the incidence of MACCE in both groups as the primary study endpoint. Using the two-sided Logrank test, the risk of MACCE (HR) in the experimental group was expected to be reduced by 25% compared with the control group, i.e., HR=0.75. The two-sided test level was set to be 0.05, the test power was 90%, the study enrollment time was expected to be 1 year, the follow-up time was 3 years, the dropout rate of both groups was 10%, the 1:1 balance design was adopted, and the sample size was estimated to be 299 participants in each group. Therefore, 300 eligible participants were planned to be included in each of the two groups, for a total of 600 participants.

**3.2 Study methods**

Multicenter, prospective, randomized, open-label, parallel-controlled clinical study.

**3.3 Study procedure**

1) Screening of patients who meet the inclusion criteria according to the inclusion criteria;

2) Communicate with patients who meet the inclusion conditions, sign the informed consent form, and arrange randomization;

3) the enrolled patients were immediately assigned to the glucose-containing dialysate group and the glucose-free dialysate group;

4) Observe and record relevant data, adverse reactions, events and treatment during hemodialysis treatment;

5) collect outcome measures for the follow-up process;

6) The data were summarized and processed, and the differences in the efficacy of glucose-containing dialysate and glucose-free dialysate in the treatment of maintenance hemodialysis patients were analyzed and compared.

|  | **V1** | **V2** | **V3** | **V4** | **V5** | **V6** | **V7** |
| --- | --- | --- | --- | --- | --- | --- | --- |
|  | **-1M** | **0M** | **3M±14d** | **6M±14d** | **12M±14d** | **24M±14d** | **36M±14d** |
| **Informed consent** | **×** |  |  |  |  |  |  |
| **medical history** | **×** | **×** | **×** | **×** | **×** | **×** | **×** |
| **Physical examination** | **×** | **×** | **×** | **×** | **×** | **×** | **×** |
| **Inclusion and exclusion criteria** |  | **×** |  |  |  |  |  |
| **Dialysis prescription** | **×** | **×** | **×** | **×** | **×** | **×** | **×** |
| **Routine blood test** | **×** | **×** | **×** | **×** | **×** | **×** | **×** |
| ***** **Routine blood biochemical examination** | **×** | **×** | **×** | **×** | **×** | **×** | **×** |
| **Blood glucose** | **×** | **×** | **×** | **×** | **×** | **×** | **×** |
| **Blood pressure** | **×** | **×** | **×** | **×** | **×** | **×** | **×** |
| **Kt/V** | **×** | **×** |  |  | **×** | **×** | **×** |
| **Dysfunction of vascular access** | **×** | **×** | **×** | **×** | **×** |  | **×** |
| **Echocardiography** |  | **×** |  |  | **×** | **×** | **×** |
| **MACCE** | **×** | **×** | **×** | **×** | **×** | **×** | **×** |
| **ICFS-10 fatigue rating scale** |  | **×** |  |  | **×** | **×** | **×** |
| **Complications** | **×** | **×** | **×** | **×** | **×** | **×** | **×** |
| **Concomitant medications** | **×** | **×** | **×** | **×** | **×** | **×** | **×** |
| **Adverse events** |  | **×** | **×** | **×** | **×** | **×** | **×** |

*Routine blood biochemical examination includes liver function (ALT, AST, ALP, Alb, TB and DB), renal function (SCr and BUN), electrolytes (blood calcium and phosphorus), blood lipids (TC, TG, LDL and HDL).

**4. Participants**

Inclusion criteria: (1) Age between 18 and 75 years; (2) Having undergoing maintenance HD three times a week for at least three months and the dialysis mode is relatively fixed; (3) Kt/v ≥ 1.2 in the last 8 weeks before entering the study; (4) Provision of informed consent prior to any study specific procedures.

Exclusion criteria: (1) Participants with life expectancy less than 1 year; (2) Participants hospitalized due to decompensation or any other complications of diabetes within 3 months; (3) Participants under active infection or active cancer treatment; (4) Receiving glucose-containing dialysates in the past 3 months or intolerance to glucose-containing dialysates; (5) Planned coronary intervention, or cardiac treatment (e.g. valve); (6) Participation in another clinical intervention trial within the last 3 months; (7) Pregnancy, or impending miscarriage; and (8) Inability of the patient to understand or comply with the study.

Withdrew Criteria: 1) The patient voluntarily withdrew from this study; 2) Those who have serious complications and/or adverse events and are judged by the investigator to be unsuitable for continuing the study.

**5. Other treatment methods available**

If there are serious adverse events during the study, the patient can withdraw from the study and continue hemodialysis treatment with the original dialysate or other regimens; If the patient requests to withdraw from the study during the course of treatment, he or she can withdraw from the study and take other treatments.

**6.Test items and test time**

1) Baseline data collection: Demographic and clinical data of the participants were collected, including gender, age, height, weight, etiology of end-stage renal disease (ESRD), age on dialysis, comorbidities (hypertension, diabetes, previous history of CVD) and other data. Prior history of CVD is defined as having had myocardial infarction, angina, percutaneous coronary intervention or coronary artery bypass surgery, chronic congestive heart failure, and cerebrovascular disease.

2) Dialysis prescription: dialysis mode, dialysis time, ultrafiltration volume, replacement amount, heparin type and dose.

3) Laboratory tests before hemodialysis: blood routine, liver function (ALT, AST, Alb, ALP, TBIL, DBIL), blood lipids (TG, TC, LDL, HDL), blood calcium, blood phosphorus, PTH, and C-reactive protein.

4) Blood glucose: All patients measured and recorded the blood glucose value (postprandial blood glucose) during the first hemodialysis per week (0 h before dialysis, 2 h during dialysis, and 0 h after dialysis) in the first 48 weeks after enrollment.

5) Blood pressure: All blood pressure values before and after dialysis were measured and recorded for all patients.

6) Vascular access dysfunction events during hemodialysis: including arteriovenous fistula (AVF)/arteriovenous graft (AVG) surgery, temporary/permanent double-lumen catheter placement, percutaneous interventional therapy, or surgical thromboembolectomy.

7) Dialysis adequacy: spKt/V.

8) MACCE: (1) new or exacerbated congestive heart failure and myocardial infarction, (2) cardiogenic shock and/or sudden cardiac death, (3) transient ischemic attack or new and exacerbated cerebral infarction or hemorrhage, (4) pulmonary embolism and peripheral arterial thrombosis or embolism.

9) Cardiac ultrasound: Measurement of left ventricular end-diastolic diameter (LVEDD), left ventricular posterior wall thickness (PWTd), ventricular septal thickness (SWTd), and left ventricular ejection fraction (LVEF). Relative wall thickness (RWT) and left ventricular mass (LVM) and left ventricular mass index (LVMI) were calculated.

10) Fatigue level: ICFS-10 fatigue rating scale.

11) Comorbidities and concomitant medications.

**7. Outcomes**

Primary outcome: the incidence of MACCE. MACCE is defined when the following conditions occur during maintenance HD: (1) Occurrence or deterioration of congestive heart failure and myocardial infarction; (2) Cardiogenic shock and/or sudden cardiac death; (3) Transient ischemic attack or occurrence and deterioration of Cerebral infarction and cerebral hemorrhage; (4) Pulmonary embolism and peripheral arterial thrombosis or embolism.

The secondary outcomes include:

(1) All-cause mortality at 1year, 2 years and 3 years follow-ups.

(2) The incidence of intradialytic hypotension (IDH). According to the National Kidney Foundation Kidney Disease Outcomes Quality Initiative (KDOQI), IDH is defined as “a decrease in systolic blood pressure (SBP) by ≥ 20 mmHg or a decrease in mean arterial pressure (MAP) by ≥10 mmHg, accompanied by hypotension symptoms such as abdominal discomfort, yawning, sighing, nausea, vomiting muscle cramps, restlessness, dizziness or fainting, and anxiety”.

(3) The incidence of hypoglycemia. A serum glucose level below 3.9 mmol/L (70 mg/dl) is defined as hypoglycemia.

(4) Blood pressure variability during HD. Systolic blood pressure (SBP) and diastolic blood pressure (DBP) will be measured before HD, every hour during HD, and after HD. Blood pressure coefficient of variation (CVBP) will be used to represent the variability of SBP and DBP during HD. CVBP = standard deviation of blood pressure / mean blood pressure.

(5) Blood glucose variability during HD. Within the first 48 weeks after enrollment, we will measure blood glucose (postprandial blood glucose) during the first HD process each week (0h before HD, 2h during HD and 0h after HD). Blood glucose coefficient of variation (CVBG) = standard deviation of blood glucose / mean blood glucose.

(6) Dysfunction of vascular access.

(7) Cardiac function. Left ventricular end diastolic diameter (LVEDD), left ventricular posterior wall thickness (PWTd), interventricular septal thickness (SWTd), and left ventricular ejection fraction (LVEF) will be measured using echocardiography. Meanwhile, we will also calculate relative wall thickness (RWT), left ventricular mass (LVM), and left ventricular mass index (LVMI).

(8) Fatigue level: ICFS-10 fatigue rating scale.

Safety evaluation: adverse event analysis, vital signs, laboratory parameters (such as blood routine).

**8. Observation, recording, and disposal of adverse events**

Definition of adverse event: Any untoward medical occurrence throughout the study period, which does not necessarily have a causal correlation with the study. Each adverse event that occurred during the course of the study should be fully recorded, including the date, time, severity, process description, measures administered, causal relationship estimation of the adverse event and related treatment, and outcome.

Serious Adverse Events: Serious adverse events (SAEs) are considered to be serious if the following adverse events occur during the course of the study: death, need for hospitalization or prolonged hospitalization, life-threatening, persistent or significant loss of function, congenital malformation or birth defect, and serious in nature from a medical point of view.

Causality of severity and adverse events:

Severity: The investigator determines the need for treatment based on clinical experience and whether treatment is required, and each adverse event should be divided into:

1) Mild: Symptoms and signs are mild, short in duration, do not require treatment, and do not affect daily life.

2) Moderate: The patient is unwell and has a mild impact on daily life.

3) Severe: It seriously affects daily life, and it is not easy to recover with simple treatment, resulting in loss of function and/or life-threatening, and even death.

Causality:

1) Likely to be related: The occurrence of adverse events has a significant temporal relationship with the study of the dialysis treatment pattern, or the recurrence of the dialysis treatment pattern and other causative factors are unlikely.

2) Possibly related: There is a significant temporal relationship between the occurrence of adverse events and the study of dialysis treatment mode, and the likelihood of other causative factors is equal to or less likely than that of dialysis treatment mode.

3) Potentially unrelated: There was no significant temporal relationship between the occurrence of adverse events and the study of dialysis treatment patterns and/or the presence of other, more likely causative factors.

4) Unrelated: Adverse events occur due to pre-existing or concurrent morbidity, or there is no temporal relationship and there is a high probability of other factors.

Follow-up of adverse events: When an adverse event occurs, the investigator should actively take appropriate measures to ensure the safety of the patient. Any AEs observed from the subject's entry into the study until the end of the study should be followed up until event resolution. Incident resolution refers to the subject's health returning to normal or baseline status or stable or lost to follow-up. If follow-up is not possible for any reason, it must be explained in the original medical record and CRF. Investigators can choose a variety of follow-up methods such as hospitalization, outpatient, home visit, and communication according to the severity of the subject's adverse reactions.

**9. Quality control and quality assurance of this study**

The research team has accumulated rich experience in clinical research design and data analysis, which provides an important guarantee for the quality of project implementation, data collection and statistical analysis in the later stage of the study. Before the start of the clinical trial, the investigator should receive training on the trial protocol, so that the researcher has a full understanding and awareness of the clinical trial protocol and the specific connotation of each index. The quality control personnel should check the basic conditions of the clinical trial to ensure that the clinical trial conditions can meet the requirements of the protocol. During the trial, the investigator should carefully perform clinical operations and other work in accordance with the requirements of the institutional SOP and test protocol, and record them in a true, timely, complete and standardized manner. The quality control personnel conduct quality verification on the test process and the corresponding original records. After the end of the test, the research unit sorts out the corresponding project documents, which are checked by the quality control personnel and archived for preservation. The quality assurance department of the clinical research unit conducts a quality check of the feasibility of the tests carried out. When non-conformities are found, the investigator and the person in charge of the unit will be notified in time to make corrections, and the corrections will be tracked.

**10. Data security audits**

The clinical study will develop a data security monitoring plan according to the size of the risk. All adverse events are recorded in detail, properly handled and tracked until properly resolved or the condition is stable, and serious adverse events and unexpected events are reported to the ethics review committee, competent authorities, sponsors and drug regulatory departments in a timely manner in accordance with regulations; The principal investigator regularly conducted a cumulative review of all adverse events, and convened investigator meetings to evaluate the risks and benefits of the study if necessary; Double-blind trials can be urgently unblinded if necessary to ensure the safety and rights of subjects.

**11. Statistical analysis**

Descriptive statistical results are expressed as mean ± SD, median and interquartile range (IQR), or percentage of all patients, as appropriate. Baseline characteristics of the two treatment groups were compared using a two-sample t-test or Pearsonχ2 for continuous and categorical variables, respectively. Linear mixed-effects models are used for the analysis of repeated measures data. The change in the outcome-adjusted mean from baseline to each follow-up period between treatment groups was estimated using linear mixed-effects analysis, with outcome, time, and treatment at baseline levels as fixed effects and patients adjusted as covariates as randomized items to determine the cause of repeated measures. Results are presented with 95% confidence intervals (CIs).

For the main indicators, the cumulative probability of MACE between the two groups was calculated by the Kaplan–Meier method, and the Logrank method was used for comparison between the groups, and then the COX regression model considering the central effect was used for multivariate statistical analysis. Other secondary indicator data shall be implemented with reference; The logistic regression model considering the central effect could be used for the two groups of continuous data (incidence). The ANOVA model considering the central effect was used for the two groups of connoviosis. The CMH method, which considered the central effect, was used for both groups of data.

Subgroup analysis: The pre-planned subgroup stratification factors in this study included the presence or absence of diabetic nephropathy and the frequency of HDF use.

All of the above hypothesis tests were tested using a two-sided test with α=0.05. Statistical analysis will be performed using SAS9.4 or SPSS 23.0 statistical analysis software.

**12. Clinical Research Ethics Principles and Requirements**

Clinical research will follow the relevant provisions of the Declaration of Helsinki of the World Medical Conference and the Measures for the Ethical Review of Biomedical Research Involving Humans of the National Health and Family Planning Commission of the People's Republic of China, and specifically implement the principles and requirements of informed consent, privacy protection, free and compensation for research, risk control, protection of special subjects and compensation for research-related damages. The clinical study is not carried out until the study is approved by the Ethics Review Committee prior to the start of the study. Before each subject is enrolled in this study, the investigator has the responsibility to give the subject or/and his legal representative a complete and comprehensive introduction to the purpose, procedures and possible risks of this study, and sign a written informed consent form, and should let the subjects know that their participation in the clinical study is completely voluntary, they can refuse to participate or withdraw from the study at any stage of the trial at any time without discrimination and retaliation, and their medical treatment and rights and interests will not be affected. The informed consent form should be retained as a clinical research document for future reference, so as to effectively protect the personal privacy and data confidentiality of the subjects.

**13. Progress of the study**

1) 2024.01-2024.09: Ethics approval of each center and research registration;

2) October 2024 - September 2025: Randomized controlled trials were conducted in each center to include relevant patients;

3) October 2025-May 2028: follow-up of the primary and secondary outcomes of the included patients, and collection of relevant clinical and laboratory data;

4) June 2028 - September 2028: Conduct statistical analysis, write articles, and conclude the topic.

**14. Key researchers**

| **Name** | **Title** | **specialty** | **Division** | **GCP certificate** |
| --- | --- | --- | --- | --- |
| Ling Zhang | Professor | Nephrology | Overall mastermind and coordination | YES |
| Zhifeng Zhou | Junior | Nephrology | Participants inclusion, data analysis | YES |
| Qing Xu | Junior | Nephrology | Participants inclusion, data analysis | YES |
| Chen Liu | Junior | Nephrology | Participants inclusion, data analysis | YES |
| Ping Fu | Professor | Nephrology | Project guidance | YES |

**15. References**

1. Gansevoort RT, Correa-Rotter R, Hemmelgarn BR, Jafar TH, Heerspink HJ, et al. Chronic kidney disease and cardiovascular risk: epidemiology, mechanisms, and prevention. Lancet. 2013;82:339–352.

2. 张宇宇,黄芸,王瑛,等. 含糖透析液对糖尿病肾病血透患者影响因素研究的Meta分析[J]. 现代实用医学,2018,30(12):1579-1581.

3. Ricks J, Molnar MZ, Kovesdy CP, et al. Glycemic control and cardiovascular mortality in hemodialysis patients with diabetes: a 6-year cohort study. Diabetes. 2012;61(3):708-715.

4. Burmeister JE, Scapini A, da Rosa Miltersteiner D, da Costa MG, Campos BM. Glucose-added dialysis fluid prevents asymptomatic hypoglycaemia in regular haemodialysis. Nephrol Dial Transplant. 2007;22(4):1184-1189. doi:10.1093/ndt/gfl710

5. Cui L, Meng Y, Xu D, et al. Analysis of the metabolic properties of maintenance hemodialysis patients with glucose-added dialysis based on high performance liquid chromatography quadrupole time-of-flight mass spectrometry. Ther Clin Risk Manag. 2013;9:417-425. doi:10.2147/TCRM.S49634

6. Abe M, Kalantar-Zadeh K. Haemodialysis-induced hypoglycaemia and glycaemic disarrays. Nat Rev Nephrol. 2015;11(5):302-313. doi:10.1038/nrneph.2015.38

7. Alsahli M, Gerich JE. Hypoglycemia, chronic kidney disease, and diabetes mellitus. Mayo Clin Proc. 2014;89(11):1564-1571. doi:10.1016/j.mayocp.2014.07.013

8. Yu TM, Lin CL, Chang SN, Sung FC, Kao CH. Increased risk of stroke in patients with chronic kidney disease after recurrent hypoglycemia. Neurology. 2014;83(8):686-694.

9. Li M, Li Y, Lv J, et al. The effects of glucose-free and glucose-containing dialysate during dialysis in MHD patients: a prospective cross-over study. Perfusion. 2023;38(1):178-185. doi:10.1177/02676591211042726

10. 丁雪梅,周光霞,辛霞. 含糖透析液对糖尿病肾病患者血液透析期间血糖影响的Meta分析[J]. 中国血液净化,2018,17(12):818-823. DOI:10.3969/j.issn.1671-4091.2018.12.007.

**生物医学伦理研究方案**

**（干预性临床研究）**

含糖透析液 vs. 无糖透析液治疗维持性血液透析患者的疗效评估：一项多中心随机对照临床试验研究**方案**

研究单位：四川大学华西医院等

项目负责人（签名）：张凌

承担科室：肾脏内科

联系电话：18980601985

组长单位：四川大学华西医院

参加单位：西南医科大学附属医院等

研究年限：2024年10月 —— 2028年5月

版本号：V1.0

版本日期：2023年12月20日

**方 案 摘 要**

| **研究设计**  **(可多选)** | **□病例对照研究 □队列研究 □横断面研究**  **■随机对照研究 □应用盲法 □其他：** |
| --- | --- |
| **研究类型**  **（请根据项目类型勾选）** | **（A类：高风险）**  □Ⅲ类临床新技术（安全性、有效性确切，技术难度大、风险高）  □ 特殊人群研究（儿童、孕妇、智力低下者、精神障碍受试者等）  □ 超药物说明书研究（□超适应症 □超给药途径 □超剂量 □超年龄  □超禁忌症 □超人群 □其他，请说明： ）  □ 超器械说明书研究（□超适应症 □使用范围 □超禁忌症 □超人群  □其他，请说明： ）  □ 其他（研究者判定，请说明： ）  **（B类：中风险）**  □ 上市后生物制剂研究（预防用和治疗用）  □ 上市后治疗性疫苗研究  □ 上市后罕见病药物研究  □ Ⅱ类临床新技术（安全性、有效性确切，有一定技术难度，有一定医疗风险和伦理风险）  □ 其他（研究者判定，请说明： ）  **（C类：低风险）**  ■已上市5年药物研究（包括化药、仿制药等）  □ 已上市器械研究（含AI，影像软件）  □ Ⅰ类临床新技术（安全性、有效性确切，技术难度低、几乎不存在伦理风险的医疗技术）  □ 其他（研究者判定，请说明： ） |
| **病例总数** | 600 |
| **风险/受益分析** | 目前关于透析液含糖浓度及作用的研究结论尚不统一，缺乏相关高级别的循证学依据。本研究观察5.5mmol/L的葡萄糖透析液对ESKD患者主要心脑血管不良事件发生率等各项指标，并观察对3年死亡率的影响，评价含糖透析液的有效性和安全性，为探索ESKD患者最佳透析治疗处方和指南的建立提供新的证据 |
| **风险判断** | □不大于最小风险 ■大于最小风险  最小风险：指试验中预期风险的可能性和程度不大于日常生活、或进行常规体格检查或心理测试的风险 |

一、研究背景

慢性肾脏病（chronic kidney disease, CKD）的患病率在全球范围内不断上升，尤其是随着糖尿病（diabetes mellitus, DM）、高血压和肥胖等非传染性疾病的患病率不断上升[1]。CKD是一种与年龄相关的肾功能下降的临床表现。CKD患病率的增加表明，相当多的CKD患者最终可能发展为终末期肾病（end stage kidney disease, ESKD），并需要肾脏替代治疗。血液透析（hemodialysis, HD）是ESKD患者中最常见的肾脏替代治疗。

现阶段大部分医疗机构的血透中心均采用无糖透析液进行透析，它的优点在于不利于细菌生长，且不容易影响血脂代谢[2]。近年来，2型糖尿病（T2DM）是ESKD的主要原因之一，血糖控制对维持性血液透析（maintenance hemodialysis, MHD）患者尤为重要。HD患者由于肾糖异生障碍、营养不良和无糖透析液而容易发生低血糖。HD患者的严重低血糖（<5.55 mmol/L）可导致癫痫发作、昏迷和死亡，增加中风风险，并增加相关的发病率和死亡率[3]。HD可独立导致HD患者的低血糖事件[4]，因为使用无葡萄糖或低葡萄糖透析液进行4小时HD时，会损失20-40 g葡萄糖[5]。

因此，透析液中加入适量的葡萄糖，能一定程度上减少透析液中糖的丢失，弥补血透过程中患者机体血糖的不足，从而有效降低低血糖的发生率；而低血糖发生率的降低，又能一定程度上减少血压降低的风险。目前，大多数欧洲国家、美国和日本使用含有葡萄糖的透析液来预防低血糖[6]。研究表明，含5-10 mmol/L葡萄糖的透析液可减少低血糖、低血压和心率变异性[7,8]。我国一项前瞻性交叉研究结果表明，使用无糖透析液和5.55 mmol/L葡萄糖透析液均会降低透析后的血糖，其中含糖透析液可以降低低血糖的发生率，降低血清钠，但对透析过程中的血压没有影响[9]。有Meta分析表明，5mmol/L或5.5mmol/L的含糖透析液既增加了透析期间糖尿病肾病患者的血糖,预防透析低血糖的发生,还避免了血糖过高和其引起的副交感神经的活动,建议条件允许的医疗单位使用与生理浓度相接近的5mmol/L或5.5mmol/L的葡萄糖透析液[9]。相反，另一项干预研究发现，在单次HD期间，使用含5.55 mmol/L葡萄糖透析液对铁调素-25的清除率较无糖透析液低，表明铁调素-25的清除可能受到透析液葡萄糖浓度的影响，含糖透析液可能不是所有MHD患者的最佳选择。

目前关于透析液含糖浓度及作用的研究结论尚不统一，各研究样本量普遍较少，且未有进一步探讨对透析患者的长期预后影响，缺乏相关高级别的循证学依据。本研究将通过多中心、前瞻、随机对照研究，观察5.5mmol/L的葡萄糖透析液对ESKD患者血糖、血压变化，血压变异度、低血糖事件、血管通路障碍事件，以及主要心脑血管不良事件发生率等各项指标，并观察对3年死亡率的影响，旨在评价含糖透析液的有效性和安全性，为探索ESKD患者最佳透析治疗处方和指南的建立提供新的证据。

二、研究目的

1. 主要目的： 评价含糖透析液治疗对维持性血液透析患者主要心脑血管不良事件（Major Adverse Cardiac and Cerebrovascular event, MACCE）的影响。

MACCE指全因死亡、心力衰竭、再发心肌梗塞、脑血管意外、反复心脑血管血运重建。

参考文献：Mäkikallio T, Holm NR, Lindsay M, et al. Percutaneous coronary angioplasty versus coronary artery bypass grafting in treatment of unprotected left main stenosis (NOBLE): a prospective, randomised, open-label, non-inferiority trial. Lancet. 2016;388(10061):2743-2752.

2. 次要目的：1) 评价含糖透析液治疗降低维持性血液透析患者全因死亡的作用；

2) 评价含糖透析液治疗对维持性血液透析患者低血压、低血糖、血管通路功能障碍、心功能、疲劳程度等的影响；

3) 评价含糖透析液治疗的安全性。

三、研究设计、方法与研究步骤

1. 研究设计

拟采用开放性、多中心平行随机对照研究的方式，拟纳入四川大学华西医院、西南医科大学附属医院等30多家医院共 600 例符合纳入标准的透析龄≥3个月，每周3次常规血液透析治疗的稳定ESRD患者进入研究，签署知情同意书后，根据是否糖尿病肾病进行分层，进行区组随机化分组，区组长度为4或6，将受试者按1：1比例随机分配到含糖透析液组和无糖透析液组，收集相关主要和次要结局指标数据，比较含糖透析液和无糖透析液治疗维持性血液透析患者的疗效差异。

透析液配方：无糖组透析液：透析液流量500 ml/min，钠138 mmol/L，钾2.0 mmol/L，钙1.25-1.75 mmol/L，碳酸氢根33.5 mmol/L，温度35.5~37℃，细菌数＜200 cfu/ml，内毒素＜2 EU/ml；含糖组透析液配方：透析液流量500 ml/min，钠138 mmol/L，钾2.0 mmol/L，钙1.25-1.75 mmol/L，碳酸氢根33.5 mmol/L，葡萄糖5.5 mmol/L，温度35.5~37℃，细菌数＜200 cfu/ml，内毒素＜2 EU/ml。

透析处方：维持性血液透析1周3次，血管通路可选择自体动静脉内瘘、带涤纶套血液透析导管及人工血管等。透析液流速为 500ml/min，血流速度 200-300ml/min，透析器推荐采用高通透析器，膜面积 1.4-1.8m2，膜超滤系数>20ml/h/mmHg。抗凝可选择普通肝素或者低分子肝素作为抗凝剂。

患者数量：本研究以两组MACE发生率为主要研究终点计算样本量。使用双侧Logrank检验方法，预期试验组MACE发生风险（HR）较对照组降低25%，即HR=0.75。设定双侧检验水准为0.05，检验效能（power）为90％，预计研究入组时间为1年，随访时间为3年，两组脱落率均为10%，1:1平衡设计，应用样本量估计专业软件PASS 15.0.5，估算出所需样本量为每组299例。故两组拟各纳入300例合格病例，共计600例。

1. 研究方法

多中心、前瞻性、随机、开放、平行对照临床研究

3. 研究步骤

1) 根据纳入标准筛选符合纳入条件的患者；

2) 对符合纳入条件的患者进行沟通，签署知情同意书，安排随机分组；

3) 将入组患者随即分配至含糖透析液组和无糖透析液组；

4）观察并记录血液透析治疗过程中的相关数据、不良反应事件和处理；

5）收集随访过程的结局指标；

6）数据进行总结和处理，分析比较含糖透析液和无糖透析液治疗维持性血液透析患者的疗效差异。

|  | **V1** | **V2** | **V3** | **V4** | **V5** | **V6** | **V7** |
| --- | --- | --- | --- | --- | --- | --- | --- |
|  | **-1M** | **0M** | **3M±14d** | **6M±14d** | **12M±14d** | **24M±14d** | **36M±14d** |
| **知情同意** | **×** |  |  |  |  |  |  |
| **病史** | **×** | **×** | **×** | **×** | **×** | **×** | **×** |
| **体检** | **×** | **×** | **×** | **×** | **×** | **×** | **×** |
| **入排标准** |  | **×** |  |  |  |  |  |
| **透析处方** | **×** | **×** | **×** | **×** | **×** | **×** | **×** |
| **血常规** | **×** | **×** | **×** | **×** | **×** | **×** | **×** |
| ***血生化** | **×** | **×** | **×** | **×** | **×** | **×** | **×** |
| **血糖** | **×** | **×** | **×** | **×** | **×** | **×** | **×** |
| **血压** | **×** | **×** | **×** | **×** | **×** | **×** | **×** |
| **Kt/V** | **×** | **×** |  |  | **×** | **×** | **×** |
| **血管通路功能障碍** | **×** | **×** | **×** | **×** | **×** |  | **×** |
| **心脏超声** |  | **×** |  |  | **×** | **×** | **×** |
| **MACCE** | **×** | **×** | **×** | **×** | **×** | **×** | **×** |
| **疲劳评分量表** |  | **×** |  |  | **×** | **×** | **×** |
| **透析方案调整** |  | **×** | **×** | **×** | **×** | **×** | **×** |
| **合并症** | **×** | **×** | **×** | **×** | **×** | **×** | **×** |
| **伴随用药** | **×** | **×** | **×** | **×** | **×** | **×** | **×** |
| **不良事件** |  | **×** | **×** | **×** | **×** | **×** | **×** |

注：肝功能（ALT、AST、ALP、Alb、TB、DB）、肾功能（透析前后BUN）、电解质（血钙、血磷）、血脂（TC、TG、LDL、HDL）

四、病例选择

1. 入选标准

1) 年龄18～75周岁，性别不限；

2) 透析龄≥3个月的稳定的维持性血液透析患者，透析模式相对固定；

3) 入选前8周Kt/v≥1.2；

4) 签署知情同意书。

2. 排除标准

1) 预期寿命＜1年者；

2) 因糖尿病失代偿或任何其他合并症住院的患者；

3) 活动性感染者或者正在进行抗肿瘤治疗；

4）过去3个月内接受过含葡萄糖的透析液，或对含葡萄糖的透析液不耐受；

5）计划进行冠状动脉介入治疗，或心脏治疗（如瓣膜手术

6) 妊娠或哺乳者；

7) 3个月内参与过临床试验或正在进行临床试验者；

8) 研究者认为不适合参加本次试验者。

3. 终止研究标准

1) 患者自愿退出本研究；

2) 出现严重并发症和/或不良事件，经研究者判断不宜继续研究者。

1. 可供选择的其他诊疗方法

若研究过程中出现严重的不良反应事件，患者可退出此项研究并原有透析液或其他方案继续进行血液透析治疗；若患者治疗过程中要求退出此项研究，可退出此研究项目并采取其余方式进行治疗。

六、检测项目与检测时点

1) 基线资料收集：收集入选者人口学及临床资料，包括性别、年龄、身高、体重、终末期肾脏病（ESRD）病因、透析龄、合并症（高血压、糖尿病、既往 CVD 病史）等资料。既往 CVD 病史定义为曾发生过心肌梗死、心绞痛、接受过经皮冠状动脉介入治疗或冠状动脉搭桥手术、慢性充血性心力衰竭、脑血管疾病等

2) 透析处方：透析模式、透析时间、超滤量、置换量、肝素种类和剂量

3) 血液透析前实验室检查：血常规、肝功能（ALT、AST、Alb、ALP、TBIL、DBIL）、血脂（TG、TC、LDL、HDL）、血钙、血磷、PTH、C反应蛋白

4) 血糖：所有患者测量并记录入组后前48周每周第1次血液透析过程中（透析前0h、透析中2h、透析结束后0h）血糖值（餐后血糖）

5) 血压：所有患者测量并记录每1次血液透析前、透析过程中每小时及结束后所有血压值

6) 血液透析过程中血管通路功能障碍事件：包括动静脉瘘（AVF）/动静脉移植物（AVG）手术、临时/永久双腔导管放置，经皮介入治疗或外科血栓栓塞切除术

7) 透析充分性：spKt/V

8) MACE：（1）充血性心力衰竭和心肌梗死的新发或加重，（2）心源性休克和/或心源性猝死，（3）短暂性脑缺血发作或脑梗死或出血的新发和加重，（4）肺栓塞和外周动脉血栓形成或栓塞。主要终点：主要心脑血管不良事件（MACE）的发生率。

9) 心脏超声：测量左心室舒张末期内径(LVEDD)、左室后壁厚度(PWTd)、室间隔厚度(SWTd)和左心室射血分数(LVEF)。计算相对室壁厚度(RWT)和左心室质量(LVM)、左心室质量指数(LVMI)。

10) 疲劳程度：ICFS-10疲劳评分量表

11) 合并症和伴随用药

七、疗效评定标准

主要评定标准：主要心脑血管不良事件（MACE）的发生率。MACE被定义为以下情况之一：（1）充血性心力衰竭和心肌梗死的新发或加重，（2）心源性休克和/或心源性猝死，（3）短暂性脑缺血发作或脑梗死或出血的新发和加重，（4）肺栓塞和外周动脉血栓形成或栓塞。

次要评定标准：

1) 全因死亡： 1年、2年、3年的全因死亡事件

2) 低血压事件发生率：按美国肾脏基金会肾脏疾病结果质量倡议指南与欧洲血液净化最佳实践指南中的定义，即透析中收缩压下降≥20 mmHg或平均动脉压下降 10 mmHg以上，并伴有低血压症状。低血压相关症状包括困倦、嗜睡、叹息、烦躁、头痛、肌肉痉挛、呼吸困难及胸背疼痛、腹痛、恶心呕吐等。

3) 低血糖事件发生率：根据 ADA、加拿大糖尿病学会及欧洲药品管理局制定的最新标准，以血糖 <3.9 mmol/L（70 mg/dl）为低血糖的诊断标准。

4) 透析中血压变异度：所有患者测量并记录每次血液透析前、透析过程中每小时及结束后所有血压值，分别求收缩压(SBP)、舒张压(DBP)的平均值和标准差(SD)，计算变异系数(CVBP)表示透析中收缩压和舒张压变异性。

5) 透析中血糖变异度：所有患者测量并记录入组后前48周每周第1次血液透析过程中（透析前0h、透析中2h、透析结束后0h）血糖值（餐后血糖），根据血糖监测结果计算血糖变异性指标，包括血糖平均值（MBG）和血糖变异系数（CVBG），其中CVBG=血糖标准差/MBG。

6) 血管通路功能障碍事件：包括动静脉瘘（AVF）/动静脉移植物（AVG）手术、临时/永久双腔导管放置，经皮介入治疗或外科血栓栓塞切除术。

7) 心功能：左心室舒张末期内径(LVEDD)、左室后壁厚度(PWTd)、室间隔厚度(SWTd)和左心室射血分数(LVEF)。计算相对室壁厚度(RWT)和左心室质量(LVM)、左心室质量指数(LVMI)等。

8) 疲劳程度：ICFS-10 疲劳评分量表

安全性评价：不良事件分析、生命体征、实验室参数（血常规等）

八、不良事件的观察、记录和处置

不良事件定义：整个研究期间发生的任何不良的医疗事件，该事件不一定与研究存在因果相关性。在研究过程中发生的每件不良事件都应完整记录，包括不良事件发生的日期、时间、严重程度、过程描述、采取的措施、不良事件与相关治疗的因果关系估计、结局。

严重不良事件：如研究过程中发生以下不良事件，则判定为严重不良事件（SAE）：患者死亡、需住院治疗或延长住院治疗时间、威胁生命、持续或显著的功能丧失、先天性畸形或出生缺陷、从医疗角度判定事件性质严重。

严重程度和不良事件的因果关系：

严重程度：研究者根据临床经验和是否需要治疗给予确定，每件不良事件应分为：

1) 轻度：症状和体征轻微，持续时间短，无需治疗，不影响日常生活。

2) 中度：患者不适，对日常生活有轻度影响。

3) 重度：严重影响日常生活，简单治疗不易恢复，导致患者功能丧失和/或生命威胁，甚至死亡。

因果关系：

1) 很可能相关：不良事件发生与透析治疗模式研究有明显的时间关系，或再进行该透析治疗模式有复发且其他致病因素可能性很小。

2) 可能相关：不良事件发生与透析治疗模式研究有明显的时间关系，且其他致病因素可能性与透析治疗模式相等或与透析治疗模式相比可能性较小。

3) 可能不相关：不良事件发生与透析治疗模式研究没有明显的时间关系和/或有另外更有可能的致病因素存在。

4) 不相关：不良事件发生由原有的或并发疾病，或没有时间关系和另外可能性很大的因素存在。

不良事件的随访

当发生不良事件时，研究者应积极采取适当的措施确保患者的安全。从受试者进入研究至研究结束时观察到的任何 AE 均应随访至事件解决。事件解决是指受试者健康恢复至正常或者基线状态或者稳定或者失访。若由于某种原因不能进行随访，必须在原始病历及 CRF 内加以解释。

研究者可以根据受试者不良反应的轻重选择住院、门诊、家访、通讯等多种随访方式。

九、研究的质量控制与质量保证

本研究团队积累了丰富的临床科研设计、数据分析等经验，为该研究后期项目实施，数据收集，统计分析的质量提供了重要保障。临床试验启动前，研究者应接受试验方案的培训，使研究人员对于临床试验方案及其各指标具体内涵有充分理解和认识。质控人员应对临床试验基本条件进行核查，确保临床试验条件能满足方案要求。试验过程中研究者应依据机构 SOP 和试验方案要求认真执行临床操作等工作，并真实、及时、完整、规范地进行记录。质控人员对试验流程以及相应的原始记录等进行质量核查。试验结束后，研究单位整理相应的项目文件，经质控人员核对后，归档保存。临床研究单位的质保部门对所开展的试验进行可实施性的质量核查。当发现不符合项时，及时通知研究者和单位负责人进行改正，并追踪改正情况。

十、数据安全监查

临床研究将根据风险大小制定相应的数据安全监察计划。所有不良事件均详细记录，恰当处理并追踪直到妥善解决或病情稳定，按照规定及时向伦理审查委员会、主管部门、申办者和药品监督管理部门报告严重不良事件与非预期事件等；主要研究者定期对所有不良事件进行累积性回顾，必要时召开研究者会议评估研究的风险与受益；双盲试验必要时可以进行紧急揭盲，以确保受试者安全与权益。

十一、统计学处理

描述性统计结果以平均值±SD、中位数和四分位间距（IQR）或所有患者的百分比（视情况而定）表示。分别使用连续变量和分类变量的两样本t检验或Pearsonχ2对两个治疗组的基线特征进行比较。线性混合效应模型用于重复测量数据的分析。使用线性混合效应分析估计治疗组之间从基线到每个随访期的结果调整平均值的变化，将基线水平的结果、时间和治疗作为固定效应，将患者作为随机项进行协变量调整，以确定重复测量的原因。结果以95%置信区间（CI）表示。

对主要指标，通过Kaplan–Meier方法计算两组MACE的累积概率，采用Logrank法进行组间比较，随后将采用考虑中心效应的COX回归模型进行多元统计分析。其他次要指标资料参照执行；两组计量资料（发生率）可以采用考虑中心效应的logistic回归模型；两组计量资料使用考虑中心效应的方差分析模型；两组等级资料使用考虑中心效应的CMH法。

亚组分析：本研究预先计划的亚组分层因素包括：是否糖尿病肾病，HDF使用频次。

以上所有的假设检验采用双侧检验取α=0.05。统计分析将采用SAS9.4或SPSS 23.0统计分析软件进行计算。

十二、临床研究伦理原则与要求

临床研究将遵循世界医学大会《赫尔辛基宣言》和中华人民共和国国家卫生和计划生育委员会《涉及人的生物医学研究伦理审查办法》等相关规定，具体落实知情同意，保护隐私，研究免费与补偿，控制风险，特殊受试者保护和研究相关损害的赔偿原则与要求。在研究开始之前，由伦理审查委员会批准该试验方案后才实施临床研究。每一位受试者入选本研究前，研究者有责任向受试者或/和其法定代理人完整、全面地介绍本研究的目的、程序和可能的风险，并签署书面知情同意书，应让受试者知道他们参加临床研究完全是自愿的，他们可以拒绝参加或在试验的任何阶段随时退出本研究而不会受到歧视和报复，其医疗待遇与权益不受影响。知情同意书应作为临床研究文件保留备查，切实保护受试者的个人隐私与数据机密性。

十三、研究进度

1）2024 年 01 月-2024 年 9月：各研究中心获得伦理批件以及研究注册；

2）2024 年 10月-2025 年 9 月：各中心纳入相关患者并进行临床随机对照试验；

3）2025 年 10 月-2028 年 5 月：对纳入患者的主要结局指标和次要结局指标进行随访，以及收集相关临床和检验数据等；

4）2028 年 6 月-2028 年 9 月：进行数据统计分析，撰写文章，结题。

十四、参加人员

| **姓名** | | **职称** | | **专业** | | **任务** | | **GCP培训证书** | |
| --- | --- | --- | --- | --- | --- | --- | --- | --- | --- |
| 张凌 | | 教授 | | 肾脏内科 | | 项目总体策划协调 | | 是 | |
| 周志锋 | | 初级 | | 肾脏内科 | | 病例纳入、数据分析 | | 是 | |
| 胥箐 | | 初级 | | 肾脏内科 | | 病例纳入、数据分析 | | 是 | |
| 刘晨 | | 初级 | | 肾脏内科 | | 病例纳入、数据分析 | | 是 | |
| 付平 | | 教授 | | 肾脏内科 | | 项目指导 | | 是 | |
| 付平 | | 教授 | | 肾脏内科 | | 项目指导 | | 是 | |

1. 主要参考文献

1. Gansevoort RT, Correa-Rotter R, Hemmelgarn BR, Jafar TH, Heerspink HJ, et al. Chronic kidney disease and cardiovascular risk: epidemiology, mechanisms, and prevention. Lancet. 2013;82:339–352.

2. 张宇宇,黄芸,王瑛,等. 含糖透析液对糖尿病肾病血透患者影响因素研究的Meta分析[J]. 现代实用医学,2018,30(12):1579-1581.

3. Ricks J, Molnar MZ, Kovesdy CP, et al. Glycemic control and cardiovascular mortality in hemodialysis patients with diabetes: a 6-year cohort study. Diabetes. 2012;61(3):708-715.

4. Burmeister JE, Scapini A, da Rosa Miltersteiner D, da Costa MG, Campos BM. Glucose-added dialysis fluid prevents asymptomatic hypoglycaemia in regular haemodialysis. Nephrol Dial Transplant. 2007;22(4):1184-1189. doi:10.1093/ndt/gfl710

5. Cui L, Meng Y, Xu D, et al. Analysis of the metabolic properties of maintenance hemodialysis patients with glucose-added dialysis based on high performance liquid chromatography quadrupole time-of-flight mass spectrometry. Ther Clin Risk Manag. 2013;9:417-425. doi:10.2147/TCRM.S49634

6. Abe M, Kalantar-Zadeh K. Haemodialysis-induced hypoglycaemia and glycaemic disarrays. Nat Rev Nephrol. 2015;11(5):302-313. doi:10.1038/nrneph.2015.38

7. Alsahli M, Gerich JE. Hypoglycemia, chronic kidney disease, and diabetes mellitus. Mayo Clin Proc. 2014;89(11):1564-1571. doi:10.1016/j.mayocp.2014.07.013

8. Yu TM, Lin CL, Chang SN, Sung FC, Kao CH. Increased risk of stroke in patients with chronic kidney disease after recurrent hypoglycemia. Neurology. 2014;83(8):686-694.

9. Li M, Li Y, Lv J, et al. The effects of glucose-free and glucose-containing dialysate during dialysis in MHD patients: a prospective cross-over study. Perfusion. 2023;38(1):178-185. doi:10.1177/02676591211042726

10. 丁雪梅,周光霞,辛霞. 含糖透析液对糖尿病肾病患者血液透析期间血糖影响的Meta分析[J]. 中国血液净化,2018,17(12):818-823. DOI:10.3969/j.issn.1671-4091.2018.12.007.
