# Supplementary material for: Glucose-containing vs. glucose-free dialysate for patients with maintenance hemodialysis: Study protocol for a multicenter randomized controlled study-GLUMO study
Source: PLoS One. 2025 Aug 11;20(8):e0330155. doi: 10.1371/journal.pone.0330155 (PMC12338788; doi:10.1371/journal.pone.0330155)
Supplement: S4 File — (DOCX) [file pone.0330155.s004.docx]

**Informed consent form**

Dear participants,

We invite you to participate in the research project " Glucose-containing vs. glucose-free dialysate for patients with maintenance hemodialysis: a multicenter randomized controlled study-GLUMO study" approved by Jafron Biomedical Co.,Ltd and West China Hospital of Sichuan University. This study will be carried out at more than 30 dialysis centers in China, including West China Hospital of Sichuan University and the Affiliated Hospital of Southwest Medical University, with an estimated 600 participants voluntarily participating. This study has been reviewed and approved by the Biomedical Ethics Review Committee of West China Hospital, Sichuan University.

1. Why are we conducting this trial?

With the increasing prevalence of diabetes, hypertension and obesity, an increasing number of patients would develop chronic kidney disease (CKD), with a considerable number eventually progressing to end-stage kidney disease (ESKD) and requiring renal replacement therapy. Hemodialysis (HD) is the most common form of renal replacement therapy for patients with ESKD. Due to issues such as impairment of renal gluconeogenesis, malnutrition, and the use of glucose-free dialysate, HD patients are more susceptible to hypoglycemia, which is associated with seizures, stroke, and higher mortality. Incorporating an appropriate amount of glucose into the dialysate can help to offset the insufficient blood glucose during HD, potentially reducing the incidence of hypoglycemia. Currently, the efficacy and safety of glucose-containing dialysate during HD remain contentious, and this study will be conducted to evaluate the efficacy and safety of 5.5mmol/L glucose-containing dialysate for maintenance HD patients, aiming to provide new insights for future dialysis prescriptions and guidelines.

1. What do you need to do if you participate in the research?

During the research process, you will be randomly assigned to the glucose-containing dialysate group and glucose-free dialysate group. And during the study period, a certain amount of blood sample needs to be drawn for efficacy and safety evaluation. Participating in this study, you do not need to undergo additional blood tests or additional testing items.

1. What are the available treatment options?

If serious adverse reactions occur during the research process, you may choose to withdraw from the study and switch to the original type of dialysate to continue hemodialysis. If you request to withdraw from this study during the treatment process, you may withdraw from this research and adopt other methods of treatment.

1. Who are not suitable to participate in research?

If you have the following situations:

(1) Life expectancy less than 1 year;

(2) Hospitalized due to decompensation or any other complications of diabetes within 3 months;

(3) Under active infection or active cancer treatment;

(4) Receiving glucose-containing dialysates in the past 3 months or intolerance to glucose-containing dialysates;

(5) Planned coronary intervention, or cardiac treatment (e.g. valve);

(6) Participation in another clinical intervention trial within the last 3 months;

(7) Pregnancy, or impending miscarriage;

(8) Unable to understand or comply with the study.

You will not be advisable to participate in this study.

1. What are the risks of participating in this research?

This study collected relevant clinical data and information from patients. During dialysis using glucose-containing dialysate, you may be at risk of hyperglycemia, hypertriglyceridemia, and decreased clearance of potassium. We will strictly adhere to operational protocols to minimize the occurrence of related risks. If there is a relevant risk, we will deal with it in a timely manner. And in this study, we will strictly monitor various indicators to reduce the occurrence of adverse reaction events. Our research group will take strict confidentiality measures to prevent the risk of patient personal information leakage.

1. What are the possible benefits of participating in research?

By participating in this study, your condition may improve. This study will provide evidence on the efficacy and safety of 5.5mmol/L glucose-containing dialysate for maintenance HD patients, and will provide valuable insights for future dialysate selection and the enhancement of dialysis treatment prescription.

1. Do you need to pay any additional fees to participate in the research?

During the research process, you are not required to pay any additional fees except for HD treatment related expenses. In addition, we will provide you with a free specialist outpatient copay once a month during this study. If there is any injury related to the study during your participation, corresponding treatment and compensation will be provided in accordance with relevant national regulations.

1. Is personal information confidential?

Your research materials will be kept at West China Hospital of Sichuan University and other centers, and researchers, research authorities, and ethics review committees can access your medical records. Any public reports related to the results of this research will not disclose your personal identity. We will make every effort to protect the privacy and personal information of your personal medical data within the scope permitted by law.

1. Do you have to participate in the research?

Participating in this study is entirely voluntary. You may refuse to participate in the study or withdraw from the study at any stage of the trial without discrimination or retaliation, and your medical treatment and rights will not be affected. If you decide to withdraw from this study, please contact your doctor for proper diagnosis and treatment of the disease.

**Participants Statement:** I have read the above introduction about this study, and my researchers have fully explained and explained to me the purpose, operational process, potential risks and benefits of participating in this study, and answered all my relevant questions. Voluntarily participate in this study.

Name of participant: 　 Date of signature:

Tel:

Name of legal representative (if available):

Date of signature: Tel:

**Doctor's statement:** I have explained the relevant details of the study to the volunteer mentioned above, and provided him/her with an original signed informed consent form. I confirm that I have provided the participants with a detailed explanation of the situation of this study, especially the ethical principles and requirements that may arise from participating in this study, such as risks and benefits, free and compensation, damages and compensation, voluntary and confidentiality.

Name of Doctor: 　 Date of signature:

Tel:

Contact number of the Biomedical Ethics Review Committee of West China Hospital of Sichuan University: 028-85422654, 028-85423237

含糖透析液 vs.无糖透析液治疗维持性血液透析患者的疗效评估：一项多中心随机对照临床试验临床研究知情同意书

尊敬的受试者

我们邀请您参加健帆生物科技集团股份有限公司批准开展的“含糖透析液 vs.无糖透析液治疗维持性血液透析患者的疗效评估：一项多中心随机对照临床试验”课题研究。本研究将在四川大学华西医院、西南医科大学附属医院等30余家医院共同开展，估计将有600名受试者自愿参加。本次问卷时长约数分钟。本研究已经得到四川大学华西医院生物医学伦理审查委员会的审查和批准。

1. 为什么要开展本项研究？

相当多的慢性肾脏病患者最终可能发展为终末期肾病，并需要肾脏替代治疗。血液透析是终末期肾病患者中最常见的肾脏替代治疗。采用无糖透析液进行透析可导致低血糖事件的发生，严重低血糖可导致癫痫发作、昏迷和死亡，增加中风风险，并增加相关的发病率和死亡率。透析液中加入适量的葡萄糖，能一定程度上减少透析液中糖的丢失，弥补血透过程中患者机体血糖的不足，从而有效降低低血糖的发生率；而低血糖发生率的降低，又能一定程度上减少血压降低的风险。然而，目前关于透析液含糖浓度及作用的研究结论尚不统一，且未有进一步探讨对透析患者的长期预后影响，缺乏相关高级别的循证学依据。本研究的目的是观察5.5mmol/L的葡萄糖透析液对终末期肾病患者血糖、血压变化，血压变异度、低血糖事件、血管通路障碍事件，以及主要心脑血管不良事件发生率等各项指标，并观察对3年死亡率的影响，旨在评价含糖透析液的有效性和安全性，为探索终末期肾病患者最佳透析治疗处方和指南的建立提供新的证据。

1. 如果参加研究，您需要做什么？

研究过程中，您将会随机分配到含糖透析液组（健帆生物科技集团股份有限公司提供，注册备案号：国械注准20213100635）和无糖透析液组（瑞鹏医疗器械成都有限公司提供，注册备案号：国械注准20173104541，批号：C222311037，后续批号可能随着不同生产批次而改变）。研究过程中，您需要在入组后前48周每周第1次血液透析过程中（透析前0h、透析中2h、透析结束后0h）测量指尖血糖（餐后血糖），测血糖费用免费。除此之外，无其他因参加研究而额外增加的检查。研究入组时间为1年，随访时间为3年，我们将对您主要心脑血管不良事件的发生等指标进行随访。参与该研究，您不需要进行额外的抽血和更多的检查项目。

3. 可供选择的诊疗方案有哪些？

若研究过程中出现严重的不良反应事件，您可退出此项研究并原有透析液或其他方案继续进行血液透析治疗；若您治疗过程中要求退出此项研究，可退出此研究项目并采取其余方式进行治疗。

4. 哪些人不宜参加研究?

如果您存在以下情况1) 预期寿命＜1年者；

2) 因糖尿病失代偿或任何其他合并症住院的患者；

3) 活动性感染者或者正在进行抗肿瘤治疗；

4）过去3个月内接受过含葡萄糖的透析液，或对含葡萄糖的透析液不耐受；

5）计划进行冠状动脉介入治疗，或心脏治疗（如瓣膜手术

6) 妊娠或哺乳者；

7) 3个月内参与过临床试验或正在进行临床试验者；

8) 研究者认为不适合参加本次试验者；

则不宜参加本研究。

5. 参加研究有哪些风险？

本研究对患者的相关临床数据和信息进行收集；使用含糖透析液透析过程中，您可能会高血糖、高甘油三酯血症和对血钾清除水平下降等风险，我们将严格遵守操作规律，尽量避免相关风险的发生，如果出现相关风险，我们将及时进行处理；本研究将严格监测各项指标，降低不良反应事件的发生；本课题组将采取严格保密措施、杜绝您个人信息泄露风险。

1. 参加研究有哪些可能的好处？

参加本项研究，您的病情有可能获得改善，本项研究还有助于评价含糖透析液的有效性和安全性，为探索终末期肾病患者最佳透析治疗处方和指南的建立提供新的证据，可以更安全有效地治疗与您具有相似病情的其他病人。

1. 参加研究需要支付有关费用吗？

参加研究过程中，您除需要支付血液透析治疗相关费用外，无需支付其他额外费用。此外，我们将在研究期间每月免费为您提供一次专家门诊挂号费；如果您出现与研究相关的损伤时，将我们依据国家有关规定提供相应的治疗与补偿。

1. 个人信息是保密的吗？

您的研究资料将保存在四川大学华西医院等各研究中心医院，研究者、研究主管部门、伦理审查委员会可查阅您的医疗记录。任何有关本项研究结果的公开报告将不会披露您的个人身份。我们将在法律允许的范围内，尽一切努力保护您个人医疗资料的隐私和个人信息。

1. 我必须参加研究吗？

参加本项研究是完全自愿的，您可以拒绝参加研究，或在试验的任何阶段随时退出本研究而不会受到歧视和报复，其医疗待遇与权益不受影响。如果您决定退出本研究，请与您的医生联系，以便妥善诊疗疾病。

受试者声明：我已经阅读了上述有关本研究的介绍，我的研究人员已向我充分解释和说明了本研究的目的、操作过程以及参加本研究可能存在的风险和潜在的获益，并回答了我所有相关问题。自愿参加本研究。

我同意□ 或拒绝□ 除本研究以外的其他研究利用我的研究资料和生物标本。

受试者正楷姓名：

受试者签名： 　　 日期：＿ ＿ ＿ ＿ 年 ＿ ＿ 月 ＿ ＿ 日

受试者的联系电话： 　　手机号：

法定代理人正楷姓名： （如适用）

与受试者关系：

法定代理人签名： 日期：＿ ＿ ＿ ＿ 年 ＿ ＿ 月 ＿ ＿ 日

需法定代理人签署的原因：

见证人正楷姓名： （如适用）

见证人签名： 日期：＿ ＿ ＿ ＿ 年 ＿ ＿ 月 ＿ ＿ 日

需见证人签署的原因：

医生声明：我已对上述参加本研究的自愿者说明了该项研究的有关细节，并且为他/她提供一份签署过的知情同意书的原件。我确认已向受试者详细解释了本研究的情况，特别是参加本研究可能产生的风险与受益、免费与补偿、损害与赔偿、自愿与保密等伦理原则和要求。

医生签名： 　 日期：＿ ＿ ＿ ＿ 年 ＿ ＿ 月 ＿ ＿ 日

医生的联系电话：

**四川大学华西医院生物医学伦理审查委员会**  **联系电话：028-85422654，028-85423237**
