# Supplementary material for: Glucose-containing vs. glucose-free dialysate for patients with maintenance hemodialysis: Study protocol for a multicenter randomized controlled study-GLUMO study
Source: PLoS One. 2025 Aug 11;20(8):e0330155. doi: 10.1371/journal.pone.0330155 (PMC12338788; doi:10.1371/journal.pone.0330155)
Supplement: Attachment — Submitted filename: Response letter.docx [file pone.0330155.s006.docx]

Dear Editor and Reviewers,

Thank you very much for allowing us to revise the manuscript entitled “Glucose-containing vs. glucose-free dialysate for patients with maintenance hemodialysis: study protocol for a multicenter randomized controlled study-GLUMO study” (ID: PONE-D-24-53906). We are very grateful to your advice for the manuscript, and those comments and suggestions are valuable and very helpful for revising and improving our paper. According to your advice, we amended the relevant part of the manuscript. The revised portion is highlighted within the manuscript by using the track changes mode in MS word. The following are our responses to the editorial comments and reviewers’ comments.

We would like to re-submit this revised manuscript to “PLOS ONE”, and hope it is acceptable for publication in the journal. We hope you are satisfied with the revised version.

Looking forward to hearing from you soon.

With kindest regards,

Sincerely yours

**To Editor’s and Reviewers’ comments:**

**To the associate editor:**

1. *Please ensure that your manuscript meets PLOS ONE's style requirements, including those for file naming.*

**Response:** Thank you very much for your feedback. We have changed our manuscript to meet PLOS ONE's style requirements, and these changes were marked in the revised manuscript.

2. *We note that you received funding from a commercial source: [Jafron Biomedical Co.,Ltd.] Please provide an amended Competing Interests Statement that explicitly states this commercial funder, along with any other relevant declarations relating to employment, consultancy, patents, products in development, marketed products, etc. Within this Competing Interests Statement, please confirm that this does not alter your adherence to all PLOS ONE policies on sharing data and materials by including the following statement: ""This does not alter our adherence to PLOS ONE policies on sharing data and materials.” Please include your amended Competing Interests Statement within your cover letter.*

**Response:** We sincerely appreciate your valuable comment. And we have carefully revised the 'Funding' and 'Competing Interests' sections as follows:

Funding: This work was supported by Jafron Biomedical Co.,Ltd. The funders had no role in study design, data collection, and analysis, decision to publish, or preparation of the manuscript. (**Page 18**)

Competing interests: Although this work was supported by Jafron Biomedical Co.,Ltd, this does not alter our adherence to PLOS ONE policies on sharing data and materials. (**Page 18**)

And we have included the amended Competing Interests Statement within your cover letter.

3. *When completing the data availability statement of the submission form, you indicated that you will make your data available on acceptance. We strongly recommend all authors decide on a data sharing plan before acceptance, as the process can be lengthy and hold up publication timelines. Please note that, though access restrictions are acceptable now, your entire data will need to be made freely accessible if your manuscript is accepted for publication. This policy applies to all data except where public deposition would breach compliance with the protocol approved by your research ethics board.*

**Response:** Thank you for pointing this out. This study was a protocol of a clinical trial, and no datasets were generated or analyzed during the current study. All relevant data from this study will be made available upon study completion. (**Page 18**) And All of the data of this trial will be recorded in the website (<https://www.smartedc.cn/>).

4. *Please include captions for your Supporting Information files at the end of your manuscript, and update any in-text citations to match accordingly.*

**Response:** Thank you very much for your helpful suggestion. We have included the captions of “Supporting Information files” at the end of your manuscript, and updated the in-text citations to match accordingly.

Supporting information (**Page 18**)

S1 File. Study Centers.

S2 File. SPIRIT Checklist.

S3 File. Original protocol approved by the ethics committee.

S4 File. Informed consent.

The multicenter, randomized controlled trial will be carried out at more than 30 dialysis centers in China (S1 File). (**Page 4**)

The study protocol will be designed in accordance with the Standard Protocol Items: Recommendations for Interventional Trials Checklist (SPIRIT)15 (SIPRIT checklist was shown in S2 File). (**Page 5**)

And the original protocol approved by the ethics committee was shown in S3 File. (**Page 5**)

After obtaining the written informed consent (S4 File). (**Page 6**)

5. *Please review your reference list to ensure that it is complete and correct. If you have cited papers that have been retracted, please include the rationale for doing so in the manuscript text, or remove these references and replace them with relevant current references. Any changes to the reference list should be mentioned in the rebuttal letter that accompanies your revised manuscript. If you need to cite a retracted article, indicate the article’s retracted status in the References list and also include a citation and full reference for the retraction notice.*

**Response:** Thank you for your valuable suggestion. We have verified that our manuscript does not cite any retracted articles and have carefully examined the reference list to confirm its completeness and accuracy. No changes to the reference list are required.

**To Reviewer #1:**

1. *Line 151. “use diabetes kidney disease (DKD) as a stratified basis…”. Please clarify and provide some more details.*

**Response:** It is really true as you suggested that we should provide more details on the randomization of participants. The detailed describe of randomization is as follows:

“After obtaining the written informed consent (S4 File), eligible patients will be randomized in a 1:1 ratio to receive either glucose-containing or glucose-free hemodialysis dialysate, with allocation determined by a centrally computer-generated randomization sequence. To ensure balance between groups, we will implement stratified block randomization using diabetic kidney disease (DKD) status as the stratification factor. Participants will be categorized into DKD and non-DKD subgroups, with each stratum undergoing separate randomization using computer-generated sequences with randomly permuted block sizes of 4 or 6 to maintain the 1:1 allocation ratio while preventing predictability in treatment assignment. Group A will receive glucose-containing dialysate for HD, and group B will receive glucose-free dialysate. Due to practical considerations, blinding of patients and treating clinicians was clinically impractical during this study.” (**Section “Randomization and blinding”, Page 6-7**)

2. *Line 217. Please list or give some examples of the expected or potential adverse events.*

**Response:** We sincerely appreciate your valuable suggestion. And we have revised the description as follows:

“Any adverse events occurring during HD, including metabolic complications (hypoglycemia, hyperglycemia) and cardiovascular events (hypotension, hypertension and arrhythmia), will be actively monitored and documented throughout the study period.” (**Section “Patient safety”, Page 9**)

3. *Line 244. The continuous variables will be presented in two different ways. Please clarify how they will determine which to take.*

**Response:** We sincerely apologize for not clarifying the presentation of continuous variables clearly. We have revised the description as follows:

“Continuous variables will be expressed as mean ± standard deviation (SD) when normally distributed with homogeneous variance; otherwise, median with interquartile range (IQR) will be reported”. (**Section “Statistical analysis”, Page 11**)

**To Reviewer #2:**

1. *Data on ultrafiltration rate during haemodialysis would be interesting.*

**Response:** We sincerely appreciate the reviewer's valuable suggestion on monitoring ultrafiltration rate (UFR) during hemodialysis. We fully acknowledge the clinical relevance of this parameter. And we will systematically document both the UFR (ml/kg/h) and total ultrafiltration volume (mL) for all study participants throughout the trial period. (**Table 1, Page 16**)

2. *It is not clear whether all patients are treated with haemodialysis only or also with haemodiafiltration. Please add this information.*

**Response:** Thank you very much for highlighting this critical aspect. Haemodiafiltration (HDF) will also be included in this study. Given the extended follow-up duration of this trial, most patients undergoing maintenance HD would inevitably undergo HDF at varying frequencies during the study period. Considering the potential impact of HDF modality on study outcomes, we will conduct a subgroup analysis stratified by HDF treatment frequency to assess its effect on result robustness. (**Section “Statistical analysis”, Page 11**)

3. *Information* *on pharmacological therapy of diabetes in patients is missing.*

**Response:** We sincerely appreciate your valuable suggestion. As you rightly pointed out, documenting pharmacological therapy for diabetes is indeed essential. Both diabetic and non-diabetic patients will be included in the study. Details regarding diabetes pharmacotherapy, including oral hypoglycemic agents and insulin regimens (Specific oral hypoglycemic agents and insulin regimens, dosage adjustments and reason for these adjustments) will all be systematically documented. (**Section “Participants”, Page 6**)
