# Supplementary material for: Glucose-containing vs. glucose-free dialysate for patients with maintenance hemodialysis: Study protocol for a multicenter randomized controlled study-GLUMO study
Source: PLoS One. 2025 Aug 11;20(8):e0330155. doi: 10.1371/journal.pone.0330155 (PMC12338788; doi:10.1371/journal.pone.0330155)
Supplement: Attachment — Submitted filename: Response letter-R2.docx [file pone.0330155.s007.docx]

Dear Editor and Reviewers,

Thank you very much for allowing us to revise the manuscript entitled “Glucose-containing vs. glucose-free dialysate for patients with maintenance hemodialysis: study protocol for a multicenter randomized controlled study-GLUMO study” (ID: PONE-D-24-53906R1). We are very grateful to your advice for the manuscript, and those comments and suggestions are valuable and very helpful for revising and improving our paper. According to your advice, we amended the relevant part of the manuscript. The revised portion is highlighted within the manuscript by using the track changes mode in MS word. The following are our responses to the editorial comments and reviewers’ comments.

We would like to re-submit this revised manuscript to “PLOS ONE”, and hope it is acceptable for publication in the journal. We hope you are satisfied with the revised version.

Looking forward to hearing from you soon.

With kindest regards,

Sincerely yours

**To Editor’s and Reviewers’ comments:**

**To the associate editor:**

1. *Please review your reference list to ensure that it is complete and correct. If you have cited papers that have been retracted, please include the rationale for doing so in the manuscript text, or remove these references and replace them with relevant current references. Any changes to the reference list should be mentioned in the rebuttal letter that accompanies your revised manuscript. If you need to cite a retracted article, indicate the article’s retracted status in the References list and also include a citation and full reference for the retraction notice.*

**Response:** Thank you very much for your feedback. We have verified that our manuscript does not cite any retracted articles and have carefully examined the reference list to confirm its completeness and accuracy. Below are the two revised references for your convenience (with adding the author group of studies):

Reference 21: “6. Glycemic Goals and Hypoglycemia: Standards of Care in Diabetes-2024. Diabetes Care 2024;47(Suppl 1):S111-s25. doi: 10.2337/dc24-S006” was changed to “American Diabetes Association Professional Practice Committee. 6. Glycemic Goals and Hypoglycemia: Standards of Care in Diabetes-2024. Diabetes Care 2024;47(Suppl 1):S111-s25. doi: 10.2337/dc24-S006” (**Page 20**)

Reference 22: “Glucose Concentrations of Less Than 3.0 mmol/L (54 mg/dL) Should Be Reported in Clinical Trials: A Joint Position Statement of the American Diabetes Association and the European Association for the Study of Diabetes. Diabetes Care 2017;40(1):155-57. doi: 10.2337/dc16-2215” was changed to “International Hypoglycaemia Study Group. Glucose Concentrations of Less Than 3.0 mmol/L (54 mg/dL) Should Be Reported in Clinical Trials: A Joint Position Statement of the American Diabetes Association and the European Association for the Study of Diabetes. Diabetes Care 2017;40(1):155-57. doi: 10.2337/dc16-2215” (**Page 21**)

**To Reviewer #1:**

1. *Thanks for addressing all the raised comments appropriately. I have no further questions or concerns on this manuscript.*

**Response:** Thank you very much for your kind approval of our work. It is your valuable suggestions that have significantly improved our manuscript.

**To Reviewer #3:**

1. *While blinding is understandably impractical for this intervention, it would be useful to further clarify who remains blinded in this study (e.g., outcome assessors, data analysts). This could be highlighted more explicitly in the Randomization and Blinding section.*

**Response:** We appreciate your valuable suggestion. As highlighted in the revised manuscript (**Section “Randomization and blinding”**), outcome assessors and data analysts will be fully blinded to group allocation throughout the study to minimize bias. We have now explicitly clarified this point in the text (**Page 7, Lines 159-160**) for greater transparency. Thank you for raising this important clarification.

2. *It is commendable that the authors have included UFR documentation. However, clarification is needed on whether UFR will be used as a covariate in statistical analysis, particularly for MACCE or IDH outcomes.*

**Response:** We sincerely appreciate your constructive feedback. In our statistical analysis, ultrafiltration rate (UFR) will be indeed included as a covariate, along with other clinically relevant factors such as patient characteristics (such as age, gender, comorbidities), vascular access and anticoagulation, in the multivariate models for the primary outcome. These adjustments will be implemented to account for potential confounding effects. (**Section “Statistical analysis”, Page 11, Lines 260-263**)

3. *The choice of ICFS-10 is appropriate, but it might be helpful to describe how this instrument has been validated in dialysis populations or to cite relevant literature supporting its use in this setting.*

**Response:** We sincerely appreciate your insightful suggestion regarding the validation of the ICFS-10 instrument in dialysis populations. Currently, there is still no consensus on how to define or measure post-dialysis fatigue (<https://pmc.ncbi.nlm.nih.gov/articles/PMC11571972/>). While we acknowledge that no studies have explicitly validated ICFS-10 in dialysis populations, its established construct validity in fatigue assessment across chronic diseases supports its applicability in this context. And to provide a more comprehensive evaluation of fatigue in dialysis patients, we will additionally incorporate the 9-item Fatigue Severity Scale (FSS) (<https://pubmed.ncbi.nlm.nih.gov/20805263/>). This approach will enhance the robustness of our findings while addressing potential limitations related to the ICFS-10’s specificity for dialysis settings. (**Page 9, Line 220**)

4. *Although no datasets are available at this stage, the authors may wish to specify how anonymized trial data will be shared upon completion (e.g., via a repository) to fully align with PLOS ONE’s open science principles.*

**Response:** We sincerely appreciate your constructive suggestion regarding data sharing. In alignment with PLOS ONE’s open science principles, we confirm that all anonymized trial data—including hemodialysis prescription records, original examination reports, and laboratory test results—will be made publicly available upon study completion. These data will be deposited in a recognized repository (SmartEDC, available at: <https://www.smartedc.cn/>). (**Section “Data Availability”, Page 18**)

5. *The role of the independent Data Monitoring Committee (DMC) is clearly stated. Adding the name or affiliation of the DMC chair (if available) would further demonstrate transparency.*

**Response:** Thank you for your valuable suggestion to enhance transparency regarding the Data Monitoring Committee (DMC). We fully agree and hereby confirm that the DMC chair for this trial was Professor Ping Fu, from West China hospital of Sichuan University. This information has now been added to the “Monitoring and quality control” section of the revised manuscript. (**Page 11, Lines 271-272**)

6. *The manuscript is generally well-written. Minor grammatical refinements could enhance clarity, for example: "the dialysis mode is relatively fixed" → "dialysis modality has remained stable"; "serum glucose concentration <3.0 mmol/L. Level 2 hypoglycemia" → likely a typo; should be "Level 3 hypoglycemia".*

**Response:** We sincerely appreciate your careful reading and constructive suggestions to improve our manuscript. We have implemented all recommended language refinements:

1. "The dialysis mode is relatively fixed" has been revised to "dialysis modality has remained stable" as suggested (**Page 6, Line 132**).
2. Regarding the hypoglycemia classification, we confirm this was indeed a typographical error. And "Level 2 hypoglycemia" has been corrected to "Level 3 hypoglycemia. (**Page 8, Line 202**)

We have also conducted additional proofreading to ensure all terminology is precise and consistent throughout the manuscript. Thank you for helping enhance the clarity of our work.
